# Knowledge, attitudes and practices related to avian influenza among poultry workers in Nepal: a cross sectional study

**DOI:** 10.1186/1471-2334-12-76

**Published:** 2012-03-30

**Authors:** Dinesh Neupane, Vishnu Khanal, Kamal Ghimire, Arja R Aro, Anja Leppin

**Affiliations:** 1 Unit of Health Promotion, Institute of Public Health, University of Southern Denmark, Esbjerg, Denmark; 2 School of Public Health, Curtin University, Bentley, Western Australia, Australia; 3 Nepal Family Health Program, Kathmandu, Nepal

## Abstract

**Background:**

Avian influenza is a considerable threat to global public health. Prevention and control depend on awareness and protective behaviours of the general population as well as high risk-groups. This study aims to explore the knowledge, attitudes and practices related to avian influenza among poultry workers in Nepal.

**Methods:**

The study was based on a cross-sectional study design, using a structured questionnaire administered in face-to-face interviews with 96 poultry workers age 15 and above from the Rupandehi district in Nepal.

**Results:**

The majority of respondents were male (80%), mean age was 35 (SD = 11.6). Nearly everybody was aware that AI cases had been detected in Nepal and that poultry workers were at risk for infection. The major sources of AI information were radio, TV and newspapers. Knowledge about preventive measures was high with regard to some behaviours (hand washing), but medium to low with regard to others (using cleaning and disinfecting procedures or protective clothing). Poultry workers who got their information from TV and newspapers and those who were more afraid of contracting AI had higher knowledge than those who did not. Being employed as compared to being an owner of a poultry farm as well as having a high level of knowledge was associated with practising more preventive behaviours. While on one hand many specific government control measures found a high degree of acceptance, a majority of study participants also thought that government control and compensation measures as a whole were insufficient.

**Conclusions:**

The study provides information about knowledge and practices regarding avian influenza among poultry workers in Nepal. It highlights the importance of targeting lack of knowledge as well as structural-material barriers to successfully build preparedness for a major outbreak situation.

## Background

In January 2009 Nepal faced the first localized outbreak of highly pathogenic avian influenza (AI) among poultry, followed by a second outbreak in another area in February of the same year [1,2], but no human cases were registered [3].

If Nepal were hit by pandemic flu as a consequence of re-assortment or adaptive mutation of the virus and ensuing full human-to-human transmissibility, consequences could be severe. Depending on the planning scenario, fatality numbers are estimated between 15,000 and 130,000 and large numbers of people in need of clinical care might be faced with a severe shortage of hospital beds [4]. Beside the expected toll on human life, a pandemic is bound to incur disastrous economic losses in a country where sectors like farming and tourism, which make a significant contribution to the overall economy, are expected to be particularly hard hit [5,6].

Prevention and control planning have to take account of the whole population, but there are subgroups which are particularly critical, such as poultry or pig farmers, who are among those first in line when it comes to risk of contracting AI. Also they are expected to be a "bridging population" in terms of cross-species sharing of viruses and of spreading the disease into their local communities [7,8].

In early 2006 the Government of Nepal established the Avian Influenza Control Project (AICP) and endorsed a Joint Health and Agriculture National Avian Influenza and Influenza Pandemic Preparedness and Response Plan (NAIIPPRP) [4,9] which placed particular emphasis on precautionary behaviours of poultry workers as well as the knowledge and attitudes which drive such practices. A mass media campaign informing about risks and motivating for protective behaviours had been started already soon after HPAI hit Asia in 2003 and was intensified after it had reached Nepal in early 2008.

Based on health-behaviour models such as the Health Belief Model [10] or Protection Motivation Theory [11] crisis communication campaigns usually have a strong focus on providing information and knowledge about risks and protective behaviours. Most evidence accumulated within the context of SARS, H5N1 and the H1N1 outbreak in 2009 is consistent with these models' assumptions about the relevance of risk perceptions and beliefs in the efficacy of protective behaviours [12-17]. Evidence on the role of knowledge about pandemic influenza, however, has been less unequivocal. While some studies have found positive effects on protective behaviours [18-20], others have failed to do so [21,22].

The objectives of the present study were 1) to identify levels of knowledge about preventive behaviours as well as actual preventive behaviours with regard to avian influenza in Nepalese poultry workers, 2) to investigate factors associated with knowing about and practising preventive behaviours against AI, among them socio-demographic characteristics, media use (health information from TV and newspapers), and experience of fear. Additionally, 3) for preventive behaviours the role of knowledge about such behaviours was investigated.

## Methods

### Participants and procedure

The study, which took place in April 2009, was based on a cross-sectional cluster survey design. After obtaining ethical approval from Research Ethics Committee of Institute of Medicine at Tribhuvan University in Kathmandu, Nepal, face-to-face interviews with the help of a standardized questionnaire were conducted with 96 poultry workers age 15 and above from the Rupandehi district, Nepal. This district was chosen because it is one of those with the highest number of poultry farms in the country (N = 186) and was therefore considered of critical importance, but no actual cases of H5N1 had appeared in this area at the time of the study. To prepare the sampling frame a list of poultry farms in Rupandehi was obtained from the District Livestock Services Office (DLSO), which runs a registry of all farms which habitually house more than 50 chickens. From this overall district list the three village development committees (VDCs) with the highest density of poultry farms (75% of all farms in the region) were chosen. At the second stage 10 farms were randomly selected from each of the three VDCs. From each of these farms, proportional to their size, between 1 and 5 poultry workers were interviewed based on a snowballing principle. Prior to the interviews verbal informed consent was obtained from participants. Sample size estimation for the proportion estimates was based on the following assumption: For a desired width of a 95% confidence interval of w = .20, an alpha-level of p = .05, an intra-cluster correlation of r = .02 and a number of m = 30 clusters, the required number in each cluster was n = 3.5 which corresponds to an estimated sample size of 107. The actual sample size obtained from the field was slightly below the estimation i.e. N = 96.

### Interview questionnaire

The interview was based on a standardized questionnaire. Interviewers read the questions to the study participants and recorded responses on an answering sheet. Questions about avian influenza were developed on the basis of a published questionnaire from a study on Italian poultry workers [23] as well as the WHO fact sheet on AI [24].

Socio-demographic information was collected for age, gender, school education and occupational status (owner of poultry farm versus paid employee). Awareness about avian influenza was assessed by asking whether cases of avian influenza among poultry had appeared in Nepal (yes/no). Perceptions of professional risk were measured by a question asking about whether particular professional groups such as poultry workers, butchers or health workers were at risk for contracting avian influenza (yes/no). Further, participants were asked from which sources they had obtained information about avian influenza, among them radio, TV, and newspapers. Also, they indicated how afraid they felt that they could contract avian influenza (five-point-answering scale from "not at all" to "very much"). Knowledge about protective behaviours was assessed by an open-format question without pre-formulated answering options. Respondents were asked to name all protective measures they knew against the danger of being infected due to work with poultry, and the interviewers recorded the answers on the interview sheet. For each behaviour correctly identified one point was assigned. Afterwards study participants were asked to indicate how often they were using the following preventive measures when dealing with poultry: washing hands with soap and water, donning gloves, face masks, boots/boots covers, putting on protective body garments, and washing and disinfecting utensils and surfaces (five-step-answering format from "always" to "never"). Attitudes towards government actions were assessed by first describing current government policies and then asking whether respondents agreed with, disagreed with or felt uncertain about these approaches. Finally, another open-format item asked about habitual actions taken when sick or dead poultry was found on the farm.

### Statistical analysis

Descriptive analyses were applied by using means, standard deviations and percentages. Multivariable analyses were performed using logistic regression. The variables included were chosen on the basis of the research questions, intending to test socio-demographic differences in knowledge and behaviour, as well as mass media use, levels of fear, and, for the model testing protective behaviours, the role of knowledge about such behaviours. For the model explaining knowledge age, gender, school education (primary level and lower versus higher than primary education) and occupational status (owner of poultry farm versus paid employee), use of visual mass media for health information (receiving versus not receiving health information from TV and newspapers) and degree of fear experience (scale from 1 "not at all" to 5 "very much") were entered into the equation. Knowledge was dichotomized as low level (knowing 0 to 2 protective behaviours) versus high level (knowing more than 2 behaviours). The same variables were entered into the regression for the second model testing associations with protective behaviours, which additionally included the variable "knowledge about behaviours" (continuous, 1-6). The maximal number of correct behaviours named was actually seven. However, as only extremely few respondents fell into this category the variable was recoded so that the last category included those who knew six and more behaviours. The number of protective behaviours used on a habitual basis (always and often) was dichotomized into low level of behaviours (maximally two behaviours practiced) versus high (more than two behaviours practiced). P-values smaller or equal to 0.05 were considered statistically significant. The analysis was performed with SPSS IBM Statistics 19.0 for Windows.

## Results

### Knowledge, fear and practices related to avian influenza

The majority of respondents was male (80%) and fell in the age bracket between 25 and 44 years (69%; M = 35; SD = 11.61). Nearly two thirds (63%) were owners of poultry farms, the others (37%) were employed workers. The size of the farms varied between approximately 50 and 30,000 poultry. Twenty eight per cent of the respondents had completed higher secondary and 29% lower secondary education, while 10% were illiterate. As the questionnaire was administered via personal interviews complete datasets were obtained for all respondents on the variables used in the analyses. Nearly everybody (97%) was aware that AI cases in poultry had been detected in Nepal. The main sources of information about AI were radio (99%), followed by TV (56%) and newspapers (33%); only one per cent had received health information from health workers. All of the respondents knew that poultry workers were among the "at-risk-groups" for being infected with avian influenza, and the majority (62%) expressed some degree of fear about AI. 44% described themselves as very afraid, while 18% said they were "rather afraid" (scale 1-5, M = 3.72, SD = 1.40). As for knowledge about protective behaviours in dealing with poultry, slightly over half (53,1%) of the respondents knew about up to two such measures and a further 26% were aware of three, while only small percentages of respondents could name four or more such practices. Clearly the best known among all measures was hand washing. Protective properties of gloves and face masks were less well known, but still were referred to by a majority. Only few, in comparison, named the protective potential of body suits, boots/boot covers or washing and disinfecting utensils and surfaces (see Table 1).

**Table 1:** AI Knowledge and preventive practices among poultry workers (N = 96)

Variables	Knowledge^1^	Practices^2^
	%	%

Wash hands with soap and water	88.5	100

Use face masks	53.1	27.1

Use gloves	68.8	30.2

Use special boots or boot covers	15.6	7.3

Use special body garments	8.3	3.1

Wash and disinfect utensils	28.1	40.6

Wash and disinfect surfaces	22.9	40.6

^1^Percentage of poultry workers naming the specific behaviour when asked to list all protective practices against AI

^2^Percentage of poultry workers indicating they were always or often using this practice

When it came to practicing these behaviours, hand washing with soap and water was the most prevalent practice as it was uniformly reported as being used "always" or "often". Use of other personal protective actions, however, seemed to be less common practice. Habitual washing and disinfecting of surfaces and utensils was reported by about 40%, customary use of gloves and face masks by slightly less than one third, and only very few stated that they did use special boots or protective body garments (see Table 1). About half (51%) of the group regularly practiced up to two such behaviours, about one third (31,2%) reported using three to four, and only 19,8% more than four of these protection measures.

Most government control measures found uniformly high acceptance. Thus, nearly everybody agreed that in case of an outbreak movements should be restricted (97%), all poultry on the respective farms or in the respective areas be culled (100%) and approval of the DLSO be required to restart a business (100%). However, there were also notable exceptions. Only 27% said they thought that the government steps being taken to prevent avian influenza outbreaks were sufficient, and only 42% thought that the compensation plans were adequate. When asked how they habitually acted when they encountered sick or dead poultry, most respondents stated that they used treatment (96%) and burial of carcasses (95%). Only very few said that when finding sick (1%) or dead poultry (4%) they were in the habit of notifying the District Livestock Services Office.

### Factors associated with knowledge about protective behaviours against avian influenza

Table 2 depicts the findings from a logistic regression analysis testing associations between socio-demographic factors, source of AI information, fear experience and level of knowledge about protective behaviours. None of the socio-demographic factors showed any relationship with knowledge. As nearly everybody reported having received information via radio, the analysis for media use compared only those who had received AI information from TV and newspapers with those who had not. Poultry workers who got their information from these visual mass media were significantly more likely to be in the group with high knowledge than those who did not. Also there was an inverse relationship between knowledge and fear, indicating that being less afraid was associated with knowing more about preventive behaviours.

**Table 2:** Logistic regression analysis: Factors associated with knowledge about preventive behaviours (< = 2 behaviours known/> 2 behaviours known); N = 96

Variables	OR	CI	P
Age	.97	.93-1.02	.193

Gender (1 = male; 2 = female)	.92	.26-3.26	.897

Education (1 = none to primary; 2 = higher than primary)	.79	.22-2.84	.724

Status (1 = farm owners; 2 = paid employees)	1.65	.48-5.57	.422

Health information from TV/newspapers (1 = no; 2 = yes)	3.97	1.28-12.27	.017

Fear of AI (1-5; very low to very high fear)	.68	.47-.98	.037

Model: -2 Log Likelihood = 116.73; *χ*^2 ^= 15.98; df = 6, p = 0.14; Nagelkerke R^2 ^= .21

### Factors associated with individual preventive behaviours against avian influenza

When it came to protective behaviours against personal infection one major significant difference occurred for occupational status, indicating that paid employees were considerably more likely to practice a larger number of preventive behaviours than owners of poultry farms. Mass media use, while associated with knowledge was not related with precautionary practices, while level of knowledge did make a difference for such behaviours (see Table 3).

**Table 3:** Logistic regression analysis: Factors associated with preventive behaviours (< = 2 habitual practices/> 2 habitual practices); N = 96

Variables	OR	CI	p
Age	1.02	.97-1.07	.455

Gender (1 = male; 2 = female)	2.46	.64-9.39	.189

Education(1 = none to primary; 2 = higher than primary)	2.59	.51-13.12	.250

Status (1 = farm owners; 2 = paid employees)	27.76	5.15-149.52	.000

Health information from TV/newspapers (1 = no; 2 = yes)	.87	.23-3.37	.845

Fear of AI (1-5; very low to very high fear)	.87	.58-1.30	.493

Knowledge of preventive practices (1-6)	1.66	1.04-2.65	.033

Model: -2 Log Likelihood 98.01; *χ*^2 ^= 35.04; df = 7; p < .001; Nagelkerke R^2 ^= .41

## Discussion

Awareness about the specific risk faced by poultry workers was uniformly high, and a majority of over 60% among respondents felt afraid of contracting AI. These findings are unsurprising given that the study was conducted shortly after the first AI outbreak in Nepal but might also to some extent reflect increased governmental campaign efforts at promoting awareness after the outbreak had occurred. Also, almost everyone knew about the importance of washing hands with soap and water, which had been the main message in the campaign. This finding is in line with studies on poultry workers in other countries which similarly found hand washing to be by far the best known practice [25,26]. Assessment of knowledge in other areas, however, unveiled distinct gaps and deficits. While still almost 70% knew about the protective capacity of gloves, only half of the sample mentioned face masks as an option and only few knew about special boots or boot covers and body suits. Also, only about one fourth named a basic procedure such as washing and disinfecting surfaces and utensils. Another study, on poultry workers in Nigeria, also reported "low" levels of knowledge about preventive behaviours [25], others, however, found distinctly higher rates for knowledge about face masks, boots covers and cleaning procedures [26] than the present study did. One reason for this discrepancy might be that open-format questions like those used in the present study generate lower knowledge scores than identification tasks. Also, the finding about low knowledge about cleaning/disinfecting could be interpreted as a tendency to perceive such behaviours as routine everyday practices instead of as extraordinary precautionary measures against AI. Yet, these gaps in knowledge raise concern and suggest that future campaigns should make additional efforts to specifically target poultry workers and - beyond hand washing - focus also on the more specific behaviours which are relevant for prevention and containment of the virus at the source, i.e. on the poultry farms.

Analysis of the factors which were associated with knowledge about protection showed that TV and newspapers, which carried a substantial part of the campaign messages in Nepal, played an important role. Those who received information about AI via TV and newspapers were able to name more preventive behaviours than those without that kind of exposure - an effect also reported by other studies [26,27]. This finding certainly suggests a beneficial effect of the Nepali mass media campaign but at the same time highlights deficits in reaching groups without access to these types of media - something to be considered for future health education efforts. Another relevant factor, which was negatively associated with level of knowledge, was fear. At first glance, this seems to suggest that a higher degree of fear leads to less knowledge due to defensive processes such as not wanting to deal with the threat and therefore searching for less information. While such an explanation cannot be excluded, another mechanism is more plausible. The focus in this case was specifically on knowledge about protective behaviours, not on knowledge about AI in general, its danger potential, transmission pathways etc. While the latter type of knowledge is likely to make people aware of risks and therefore also more concerned, knowledge about effective protective behaviours might rather reduce fear by creating expectancies about successful control.

The data on protective behaviours showed that washing hands with soap and water were fairly standard practice. High-frequency cleaning and disinfecting, however, was not and neither was habitual use of personal protective equipment. Low usage rates for protective clothing have recently also been reported by studies with Nigerian poultry farmers [25,26], while findings from an Italian study registered considerably higher rates [23], which probably reflects different financial resources to fund such equipment on a regular basis.

The relevance of economic constraints was also indicated by the findings from the multivariable models. There was a substantial difference in usage rates of protective equipment between poultry farm owners and employees. The latter had higher odds to use personal protective equipment than owners of farms. Employed poultry workers in Nepal tend to work more often in larger-scale, economically better-off poultry businesses whereas many owners operate small-scale family businesses. Paid employees might thus more often have been provided with protective equipment by farm management while owners of small-scale family businesses were more likely to save on expenses, thereby trading off possible longer-term preventive gains against more immediate economic savings [26,28].

The finding that those who had more knowledge were also those who actually acted more preventively is consistent with some other studies from the field [18-20] even if the overall evidence on this issue is still inconsistent [12]. One possible explanation for such discrepancies is that effects might depend upon the specific type of knowledge measured. Knowledge about effective behaviours, which was the focus of the present study, is particularly likely to enable perceptions about efficacy of behaviours which have consistently been linked to precautionary practices [12]. Nevertheless, knowledge about a threat and potential countermeasures alone will most often be insufficient to achieve behaviour change, as other factors such as economic concerns or social norms are essential for enabling or disabling such change. Yet, the findings emphasize the role of awareness-building about the availability of preventive options as a first step in generating preventive habits.

A result which raises concern is that despite a generally voiced agreement with governmental emergency control measures and bio-security regulations large parts of the respondents expressed doubts with regard to the sufficiency of such control measures and eventual compensation mechanisms. Also, in response to a question about what they habitually did in case of sudden chicken deaths only four per cent reported actually having notified authorities in case of sick/dead poultry. Similar findings have been published by other studies [29-34]. Anticipated financial losses due to culling without sufficient compensation, lack of knowledge about how to proceed in notifying authorities, but also social considerations, such as stigma and shame might play a role and need dealing with to overcome avoidance of timely reporting [35-37]. If early notification is a key component of prevention and rapid response, trust in government actions, including compensation measures, is crucial in order to enable pervasive compliance with drastic and economically threatening actions like mass culling in an outbreak situation [34].

### Limitations of the study

A major limitation of the study lies in the small, non-random sample which restricts possibilities to generalize findings from the present data and also, due to lack of power, might have led to underestimation of potential effects. Another clear weakness is the cross-sectional study design which prohibits drawing causal conclusions about the relationships between some of the variables, such as fear and knowledge or knowledge and practices. Finally, self-report on practices are generally vulnerable to recall bias and social desirability tendencies. The face-to-face-interview situation, while enabling full response-rates on all variables as well as participation of poultry workers who lack reading or writing abilities, might have additionally heightened this type of bias in assessing attitudes and behaviours. As for attitudes, however, the very low percentage of respondents reporting compliance with notification procedures indicates that such tendencies were not pervasive.

## Conclusions

The study points to issues that warrant attention in future prevention and preparedness efforts against AI. While it corroborates the relevance of cognitive factors, such as providing knowledge about effective protection measures, it also particularly highlights the important role of material resources which enable poultry workers in a low-resource country such as Nepal to put knowledge into practice. Beyond large-scale mass education campaigns, future efforts should focus more strongly on target-group-specific information and practical trainings with regard to protective behaviours, but might also consider subsidized social marketing of protective equipment.

## Competing interests

The authors declare that they have no competing interests.

## Authors' contributions

VK, KG and DN conceived of the study and participated in its design and the data collection. DN and AL performed the data analysis. DN, AL and ARA drafted the manuscript. All authors read and approved the final version of the manuscript.

## Pre-publication history

The pre-publication history for this paper can be accessed here:

http://www.biomedcentral.com/1471-2334/12/76/prepub
